# Using an experimental medicine model to understand the antidepressant potential of the *N*-Methyl-D-aspartic acid (NMDA) receptor antagonist memantine

**DOI:** 10.1177/0269881112446535

**Published:** 2012-11

**Authors:** A Pringle, E Parsons, LG Cowen, SF McTavish, PJ Cowen, CJ Harmer

**Affiliations:** Department of Psychiatry, University of Oxford, Oxford, UK

**Keywords:** Memantine, glutamate, NMDA receptor, depression, anxiety, healthy volunteer, emotional processing

## Abstract

There is growing interest in the role of the glutamatergic system both in depression and as a novel target for treatments. Preclinical studies suggested that the non-competitive *N*-Methyl-D-aspartic acid **(**NMDA) receptor antagonist memantine might have antidepressant properties, but a randomised controlled trial failed to support this. A healthy volunteer model of emotional processing was used to assess the neuropsychological profile of action of memantine. Healthy volunteers (*n*=32) were randomised to receive a single dose of memantine (10 mg) or placebo, and subsequently completed a battery of tasks measuring emotional processing, including facial expression recognition, emotional memory, dot-probe and emotion-potentiated startle tasks, as well as working and verbal memory. Memantine treated volunteers showed an increased emotion-potentiated startle, and a reduced bias for negative items in emotional recognition memory. There were no effects of the drug on any other aspect of emotional or non-emotional information processing. These results suggest that a single dose of memantine produces an early anxiogenic response in the emotion-potentiated startle similar to that seen following a single dose of the selective serotonin reuptake inhibitor, citalopram. However, the overall profile of effects is more limited than that which might be expected in response to a conventional antidepressant.

## Introduction

Although the monoamine hypothesis remains dominant in biological explanations of depression, there is growing interest in the role of the glutamatergic system in the pathophysiology of depression, and also in the possible antidepressant effects of glutamate-modifying drugs (Sanacora et al., 2008). Glutamate is the main excitatory neurotransmitter in the brain, and it is known to be important in synaptic plasticity, learning and memory. Evidence from diverse sources suggests that glutamate abnormalities may be a feature of depression (see Sanacora et al., 2012).

In terms of treatment, the high affinity *N*-methyl-D-aspartic acid (NMDA) receptor antagonist ketamine has been shown to produce a rapid antidepressant response in the treatment of refractory depression (Berman et al., 2000; Zarate et al., 2006a). Such results are compelling and have been suggested to reflect rapid correction of aberrant plasticity mechanisms in depression. However, since ketamine is associated with a number of harmful physical and psychological side effects (see Morgan and Curran, 2012) and is subject to misuse, it is unlikely to represent a practical long-term treatment approach for depression. Therefore, the development of new candidate drugs acting at glutamatergic receptors with improved tolerability and side effect profiles is needed to more fully explore this novel avenue for treatment.

Memantine is a low-moderate affinity, non-competitive, open channel NMDA receptor antagonist currently licensed for the treatment of moderate to severe Alzheimer’s disease (Reisberg et al., 2003). Animal models suggested that memantine might have antidepressant properties (Reus et al., 2010; Rogoz et al., 2002; Skuza and Rogoz, 2003), and this is supported by a successful open-label trial in depressed patients (Ferguson and Shingleton, 2007). However, a randomised controlled trial found no evidence that memantine was more effective than placebo in treating depression (Zarate et al., 2006b).

A novel model of antidepressant drug action based on changes in behavioural measures of emotional processing in healthy volunteers has recently been proposed (Harmer, 2010; Harmer et al., 2009; Pringle et al., 2011a). This model suggests that antidepressants may work by reversing negative affective biases (i.e. the tendency to preferentially process and remember negative as opposed to positive information) in depression; such biases are believed to play a critical role in maintenance of this disorder. Consistent with this, a number of studies have found reliable effects of conventional antidepressant treatment on these measures (for a recent review see Harmer, 2010). For example, a single dose of the noradrenaline reuptake inhibitor, reboxetine, increased positive affective memory recall and improved the recognition of happy facial expressions in healthy volunteers (Harmer et al., 2003b). In a similar manner, the selective serotonin reuptake inhibitor (SSRI) citalopram has been shown to result in an attentional bias to positive words (Browning et al., 2007) and increased recognition of happy facial expressions (Harmer et al., 2003a). Studies of this type suggest that antidepressant treatment results in changes in emotional processing in the absence of subjective changes in mood after both acute and subchronic (seven day) treatment. It has been suggested that considering the effects of antidepressant treatment in healthy volunteers may be useful in characterising their neuropsychological profile of action (e.g. Harmer et al., 2009).

Given the ambiguity in the role of glutamatergic processes in depression and specifically the effects of memantine treatment, the current study was designed to further characterise memantine actions on emotional processing relevant to antidepressant drug activity. Healthy volunteers were therefore randomised to receive a single dose of memantine (10 mg) or placebo in a double-blind between-groups design. Both previous trials in depressed patients titrated up to mean doses of around 20 mg (Ferguson and Shingleton, 2007; Zarate et al., 2006b). In the present acute study however, in consideration of the recommendation that memantine dosage should be titrated and the non-selectivity of memantine at higher doses (Johnson and Kotermanski, 2006), a dose of 10 mg was selected. Given the critical role of glutamate in learning and memory, we also included tasks to explore changes in working and verbal memory. These were included to assess any relationship between emotional and cognitive effects.

## Method

### Participants

Thirty-two healthy volunteers provided written informed consent and participated in the study which was approved by the local ethics committee. One volunteer from the drug group was withdrawn from the study because of an adverse reaction (vomiting), leaving a total sample of 31 volunteers (15 female; mean age 24.88 years; range 18–32 years). Participants had taken no psychotropic medication for the previous three months and were screened to be free of current or past Axis 1 disorder on the structured clinical interview for DSM-IV (First et al., 1996). All volunteers were judged to be healthy on the basis of a physical examination (including, as a minimum, measurement of vital signs, auscultation of the heart and chest, abdominal palpation and brief neurological examination) and medical history.

### Procedure

Participants came to the laboratory at 9 am and were randomly allocated to a double-blind intervention of a single dose of either memantine (10 mg) or placebo capsules. Prior to treatment participants completed the National Adult Reading Test (NART) (Nelson, 1982) and the State Trait Anxiety Inventory (STAI) (Spielberger et al., 1970). Both prior to taking their capsule, and five hours after, participants completed the Beck Depression Inventory (BDI) (Beck et al., 1961), Befindlichkeits Scale (BFS) (Von Zerseen et al., 1974) to measure changes in mood and energy and Positive and Negative Affect Scale (PANAS) (Watson et al., 1988). Dosing took place at 9.30 am and participants rested quietly for five hours before psychological testing began at 1.30 pm. During this absorption time participants were offered a snack of a small sandwich at 12.00 pm and were able to drink water freely.

### Emotional task battery

#### Emotion-potentiated startle task (EPST)

##### Stimuli

Sixty-three pictures of three categories (pleasant, unpleasant, neutral) were taken from the International Affective Picture System (gender-specified, Larson et al., 2000). Each picture was presented for 13 s (mean intertrial interval=13 s) on a computer screen. The pictures were presented in three blocks in a fixed order such that no two pictures in the same category would appear successively.

##### Procedure and recording

The eye-blink component of the startle response was recorded from the orbicularis oculi using an electromyography (EMG) startle response system (San Diego Instruments, California, USA). Acoustic probes were 50 ms (95 dB) bursts of white noise with a nearly instantaneous rise time (generated through the noise generator and amplifier of the EMG startle response system) and were delivered binaurally through headphones at 1.5, 4.5 or 7.5 s following picture onset. To minimise expectation, startle probes were skipped from two trials per valence per block, and three probes were given within the inter-trial interval. A practice session presenting nine neutral pictures and startle probes was used in the beginning to habituate participants to the startle probes. EMG signals were filtered (low cut-off, 0.5 Hz; high cut-off, 100 Hz) and rectified. Eye-blink reflex magnitudes in microvolts were calculated by subtracting the amount of integrated EMG at reflex onset from the first peak amplitude of integrated EMG between 20 and 120 ms following probe onset. Trials with no traceable eye-blink reflex were assigned a magnitude of zero and included in the analysis. Trials which were excessively noisy during the 20 ms, pre-startle baseline period were excluded. This task provides a measure of the relative acoustic startle response during unpleasant, pleasant and neutral pictorial stimuli presentation. Therefore, eye blink reflex magnitudes were *z*-transformed within subjects to allow comparison between these different conditions and to minimise inter-subject variability. Of the 31 volunteers, five were not included in the analysis because of electrode interference.

#### Emotional categorisation and memory

Sixty personality characteristic words selected to be extremely disagreeable (e.g. domineering, untidy, hostile) or agreeable (cheerful, honest, optimistic) (taken from Anderson, 1968) were presented on the computer screen for 500 ms. These words were matched in terms of word length, ratings of frequency and meaningfulness. Participants were asked to categorise these personality traits as likable or dislikable, as quickly and as accurately as possible. Specifically, they were asked to imagine whether they would be pleased or upset if they overheard someone else referring to them as possessing this characteristic, so that the judgement was in part self- referential. Classifications and reaction times for correct identifications were computed for this task.

Immediately after completion of the categorisation task, participants were asked to recall and write down as many of the personality trait words as possible, and they were given 2 min to do this. This task therefore allowed the assessment of incidental memory for positive and negative characteristics. Accuracy and false alarms for positive compared to negatively valenced stimuli were calculated. Recognition memory was then assessed by asking volunteers to respond with a ‘yes’ or ‘no’ to each item on a list containing the 60 targets plus 60 matched distractors (30 positive, 30 negative). Accuracy, reaction times and false alarms were calculated. Data from one participant was removed from the recognition task, as their performance indicated that they had misunderstood the instructions.

#### Facial emotion recognition task

The facial expression recognition task featured six basic emotions (happiness, surprise, sadness, fear, anger and disgust) taken from the Pictures of Affect Series (Ekman and Friesen, 1976), which had been morphed between each prototype and neutral (Young et al., 1997). Briefly, this procedure involved taking a variable percentage of the shape and texture differences between the two standard images 0% (neutral) and 100% (full emotion) in 10% steps. Four examples of each emotion at each intensity were given (total of ten individuals). Each face was also given in a neutral expression, giving a total of 250 stimuli presentations. The facial stimuli were presented on a computer screen (random order) for 500 ms and replaced by a blank screen. Volunteers made their responses by pressing a labelled key on the keyboard. Participants were instructed to classify each face as being one of either angry, disgusted, fearful, happy, sad, surprised or neutral, as quickly and as accurately as possible. Accuracy, reaction time and misclassifications were measured in this task.

#### Dot probe task

In this task social-threat negative word and positive words were paired with neutral words. Attentional bias was measured by recording reaction times to a probe which could be presented either behind the emotional or the neutral word. Both an unmasked and masked (word pair presented for 14 ms followed by a mask for 186 ms) conditions were presented (for a full description of the task see, for example, Horder et al., 2009). Mean reaction time and accuracy scores were recorded. To simplify these results, attentional vigilance scores were calculated for each participant by subtracting the reaction time from trials when probes appeared in the same position as the emotional word (congruent trials) from trials when probes appeared in the opposite position to the emotional word (incongruent trials).

### Cognitive tasks

#### N-back working memory task

This was a letter variant of the n-back task (Harvey et al., 2005). Working memory load was manipulated by using three levels of complexity: 1-, 2-, 3-back tasks. Briefly, volunteers were requested to indicate whether a letter presented on the screen (the ‘target’ stimulus) matched a previously presented letter (the ‘cue’ stimulus). To minimise visual and phonological strategies, only phonologically closed letters presented in upper and lower case were used. Thus, only the following characters were presented: b, B, d, D, g, G, p, P, t, T, v, V. Volunteers were instructed to ignore the case of letters and respond by pressing the space bar when the current stimulus matched the relevant previous stimulus. Volunteers also performed a control task (0-back) during which they were required to respond to a prespecified letter (x, X). All blocks consisted of a sequence of 10 consonants varying in case. Letters were presented for 500 ms with a fixed interstimulus interval of 1500 ms. Prior to each task block, an instruction screen (0-, 1-, 2-, 3-back) was presented for 2000 ms. A 4000 ms blank screen separated the instruction from the onset of the first letter. Task blocks were separated by 1000 ms of fixation cross. Four blocks of each condition were presented in a fixed pseudorandom order (0-, 1-, 2-, 3-, 1-, 3-, 2-, 0-, 2-, 1-, 0-, 3-, 1-, 0-, 3-, 2-back). All conditions were matched for the number of target and upper/lower case letters presented. Accuracy and latency were recorded. Of the 31 volunteers, four were not included in this analysis as their performance indicted that they had misunderstood the task instructions.

#### Rey Auditory Verbal Learning Task

Declarative verbal memory was measured with the Rey Auditory Verbal Learning Test (AVLT) (Rey, 1964). This measure consists of five presentations with recall of a 15 word list (acquisition), one presentation of a second 15 word list, a sixth recall trial of the original list (short delay) and a second long delay recall following a non-verbal filler task. Finally there is a recognition task, consisting of the words from the first and second lists as well as 30 other filler words, in which participants are required to correctly identify only those words from the first list. Number of words corrected recalled for each of the acquisition trials and for the short and long delay trials were recorded, as were number of words correctly recognised and false alarms in the recognition task.

### Statistical analysis

The demographic and baseline characteristics of the two groups were compared using one way analysis of variance (ANOVA). The repeated measures mood and energy scales were compared between the two groups using repeated measures ANOVA with treatment as the between-subject factor and time as the within-subject factor. Data from the facial recognition task, the dot probe, emotional categorisation and emotion-potentiated startle were analysed using repeated measures ANOVA with treatment group as the between subject factor and stimulus valence as the within subject factor. For the dot probe, masking was also a within subject factor. Statistically significant interactions were followed up with simple main effects analysis. Finally, data from the emotional memory tasks were analysed using ANOVA. Data from the acquisition phase of the AVLT were analysed using a repeated measures ANOVA with treatment group as the between-subjects factor and trial number (1–5) as the within-subjects factor, the short and long delay recall as well as the recognition variables were analysed using ANOVA with a between-subjects factor of treatment group. A sensitivity analysis was also performed to consider the statistical power of the study based on previous data exploring the effect of the SSRI, citalopram (Harmer et al., 2003a). Using recognition of happy facial expressions, this analysis showed that, with *n*=31, the study had greater than 80% power to detect an effect of similar magnitude to citalopram (G*Power 2.0).

## Results

### Baseline measures

The groups were well matched in terms of age (placebo: mean=22.20, SD=3.67; memantine: mean=24.88, SD=4.94, *p*=0.62) and verbal IQ as measured by the NART (placebo: mean=116.01, SD=2.80; memantine: mean=116.59, SD=5.00, *p*=0.70). There were no between groups differences in terms of baseline depressive symptoms as measured by the BDI (placebo: mean=2.07, SD=2.22; memantine: mean=1.19, SD=1.42, *p*=0.24), trait anxiety as measured by the STAI (placebo: mean=31.07, SD=6.12; memantine: mean=28.88, SD=5.45, *p*=0.30).

#### Mood and energy scales

Comparing measurements taking just prior to and five hours after treatment, there was no effect of memantine on depressive symptoms as measured by the BDI or mood and energy as measured by the BFS or PANAS (all *p* values>0.1, Table 1).

**Table 1.** Mood and energy over time.

		Time 1	Time 2
BDI	Placebo	2.07 (2.52)	1.47 (2.00)
	Drug	1.19 (1.42)	0.75 (1.18)
BFS (mood)	Placebo	8.27 (6.79)	8.27 (6.22)
	Drug	7.40 (7.27)	10.00 (8.63)
BFS (energy)	Placebo	3.2 (3.75)	4.00 (3.72)
	Drug	3.56 (4.99)	7.13 (6.37)
PANAS (positive)	Placebo	30.2 (5.96)	29.87 (5.41)
	Drug	28.75 (7.04)	26.56 (7.29)
PANAS (negative)	Placebo	11.87 (1.85)	10.73 (1.79)
	Drug	10.81 (1.22)	11.5 (2.42)

BDI: Beck Depression Inventory; BFS: Befindlichkeits Scale; PANAS: Positive and Negative Affect Scale. Data are mean (standard deviation).

### Emotional task battery

#### Emotion-potentiated startle

**Figure 1.**
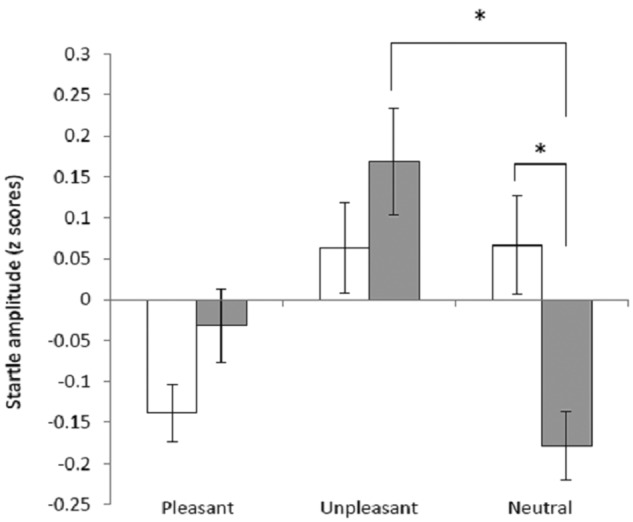
Emotion-potentiated startle. Figure shows the mean *z-*transformed eye blink response to a burst of loud white noise during pleasant, unpleasant and neutral emotional pictures for placebo (white bars) and drug (grey bars) treated groups. Error bars show standard error. * *p*<0.001.

There was an interaction between emotional valence of picture and group on both the *z*-transformed startle responses (*F*(2,48)=5.21, *p*=0.01) and the raw scores (*F*(2,48)=3.741, *p*=0.03), (Figure 1).

Simple main effects analysis showed that the emotion-potentiated startle effect was larger in the participants who received memantine (*F*(2,23)=6.24, *p*<0.01) versus placebo (*F*(2,23)=4.30, *p*=0.03). Thus, those receiving memantine showed a larger startle response to the unpleasant vs neutral stimuli compared to those receiving placebo (see Figure 1 for pairwise comparisons).

#### Emotional categorisation and memory

**Figure 2.**
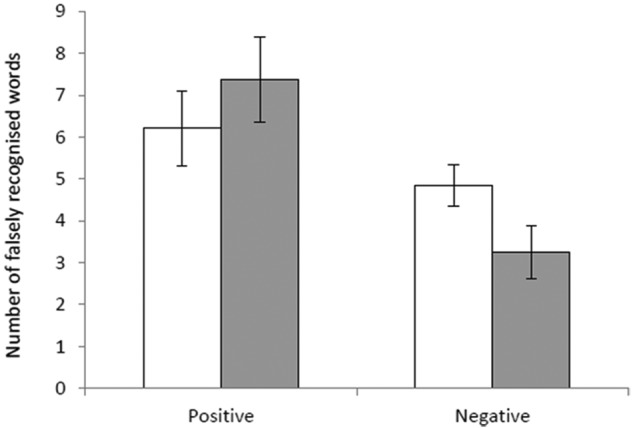
Emotional recognition memory false alarms. Figure shows the mean number of falsely recognised emotionally positive and negative personality characteristic words for placebo (white bars) and drug (grey bars) treated groups. Error bars show standard error.

There were no between-group differences in the speed to categorise self- referent personality characteristics in terms of an effect of group (*F*(1,29)=0.32, *p*=0.58) or group x emotional valence interaction (*F*(1,29) <0.01, *p*=0.95). However, there was a trend for the memantine group to make fewer negative false alarms in the recognition memory task (Group x valence interaction: *F*(1,28)=4.20, *p*=0.05, simple main effect of group for negative false alarms: *F*(1,28)=3.83, *p*=0.06, Figure 2). Memantine did not affect performance in terms of accuracy or reaction times in the recognition task or accuracy in the recall task (all *p* values >0.3).

#### Facial expression recognition

There were no effects of memantine on this task in terms of accuracy, misclassifications or reaction times (all *p* values >0.1).

#### Dot probe

**Figure 3.**
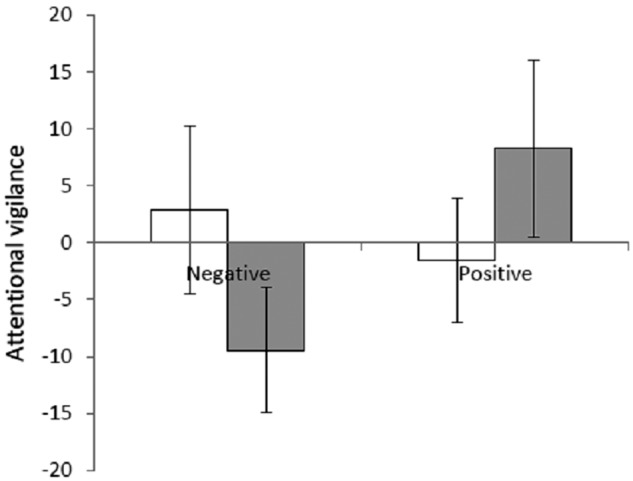
Emotional dot probe. Figure shows the mean attentional vigilance score (averaged across the masked and unmasked conditions of the task) for the placebo (white bars) and drug (grey bars) treated groups. Attentional vigilance scores were calculated for each participant by subtracting the reaction time from trials when probes appeared in the same position as the emotional word (congruent trials from trials when probes appeared in the opposite position as the emotional word (incongruent trials)). Error bars show standard error.

Including mask as within-subjects factor the mask x emotional valence x group interaction did not reach significance (*F*(1,29)=1.14), *p*=0.30) nor was there a mask x group interaction (*F*(1,29)=1.05, *p*=0.31). Collapsing across masking conditions did not affect these results (emotional valence x group interaction: *F*(1,29)=2.32, *p*=0.14, Figure 3).

### Cognitive tasks

#### N-back

There were no between-groups differences in either accuracy or latency on the control task (both *p* values >0.4). As expected, there was an effect of complexity on both accuracy and latency (main effect of complexity: *F*(2,50)=27.34, *p*<0.01 (accuracy); *F*(2,50)=12.48, *p*<0.01(latency)). However, there were no between-groups differences on these variables (both *p* values >0.8), nor was there an interaction between complexity and treatment group for either accuracy or latency (both *p* values >0.1)

#### AVLT

Memantine did not affect performance on the AVLT (all *p* values >0.3)

## Discussion

The current data suggest that acute administration of memantine has limited effects on emotional processing in healthy volunteers. Specifically, volunteers treated with memantine showed an increased emotion-potentiated startle effect and reduced bias for negative items in the emotional recognition memory task when compared to placebo. However, there were no effects of the drug on other aspects of emotional memory or emotional categorisation, the dot probe task or on the recognition of facial expression of emotion. In addition, memantine, did not affect working or declarative memory as measured by the n-back task and AVLT respectively.

The potentiated startle task is sensitive to anxiety and to anxiolytic drug treatments in both rodent and human models. SSRI treatments have been reported to initially increase the emotion-potentiated startle effect with acute administration but this effect was reversed after seven daily treatments (Browning et al., 2007; Grillon et al., 2007; Harmer et al., 2004). Such a pattern has been related to increased anxiety and agitation at the start of treatment in patient populations (Kent et al., 1998) and a similar effect can be modelled in rodent studies (Burghardt et al., 2004). There have been relatively few studies which have considered the effects of acute memantine dosing on anxiety-related processes. One study reported that low-moderate doses of the drug increased anxious responses in a mouse model of anxiety based on maternal separation distress (Takahashi et al., 2009), however, an earlier study in rats found no effect of memantine on performance on the plus maze or vogel conflict tests (Karcz-Kubicha et al., 1997). After repeated doses, preclinical studies suggest that memantine has an anxiolytic effect (e.g. Minkeviciene et al., 2008). Glutamate mechanisms including NMDA receptors are known to play a role in fear-potentiated startle in animals (Davis, 2006), and in mice, memantine increased the acoustic startle response while diminishing prepulse inhibition (Nakaya et al., 2011). Thus an action of memantine at NMDA receptors may well explain the increase in emotion-potentiated startle response seen in the present study. Clinically, there are hints that memantine may be helpful in the treatment of obsessive compulsive disorder; however, there is no evidence yet of utility in other anxiety disorders (Feusner et al., 2009).

There was a marginal effect of memantine to reduce false alarms for negatively valenced words in the emotion recognition task. The trend was seen in the absence of any effects on the two tests of non-emotional memory (n-back and AVLT) providing some evidence to suggest that it is not secondary to broader changes in memory function. Within the neuropsychological model of antidepressant drug action, emotional memory effects have usually been found in surprise free-recall tests as opposed to recognition tests, although effects on recognition memory have also been seen in some studies (Malcolm et al., 2009; Pringle et al., 2011b). However, this trend to an effect was in the absence of any other significant effects on emotional memory or categorisation. Moreover, in the present study, there were no effects of the drug on the dot probe task or the facial expression recognition task which have previously been reported to be affected by a single dose of an effective antidepressant. For example, both reboxetine and citalopram increased the perception of happy facial expressions (Harmer et al., 2003a; Harmer et al., 2003b), and reboxetine also speeded responses to positive stimuli in the emotional categorisation task (Harmer et al., 2003b).

Memantine also had no effect on memory function as measured by the n-back and AVLT. The role of glutamate in learning and memory is well-documented, and memantine is licensed for the treatment of moderate to severe Alzheimer’s disease (Witt et al., 2004). In healthy volunteers, however, memantine has been reported to disrupt recognition memory (Rammsayer, 2001) and to impair the acquisition of classical eyeblink conditioning (Schugens et al., 1997). Animal studies also report cognitive disruption in response to memantine (Dix et al., 2010; Smith et al., 2011). This is in contrast to the positive effects have been reported in preclinical models of cognitive impairment (Minkeviciene et al., 2004), and of course to the improvements in cognition reported in clinical trials of Alzheimer’s disease (Mecocci et al., 2009). This discrepancy may be explained by differences in glutamatergic state or tone (Parsons et al., 2007). A plausible explanation for the lack of effect on memory here is the relatively low dose (10 mg) which was employed in the present study, compared to the 30 mg dose used in previous studies demonstrating deleterious effects of the drug (Rammsayer, 2001; Schugens et al., 1997). The dose of 10 mg was selected here for two reasons, firstly because it is recommended that memantine dosage be titrated up to avoid unwanted side effects, and secondly because at higher doses (resulting in cerebrospinal fluid concentration so 10–500 µM) the drug is non-selective, with additional targets being affected including serotonin and dopamine uptake, nicotinic acetylcholine receptors, serotonin receptors, sigma-1 receptors and voltage activated Na^+^ (Johnson and Kotermanski., 2006). Future studies are required to assess whether stronger effects on both emotional processing and cognition are seen with repeated administration of clinically used memantine doses. Future studies will also need to control for family history of alcohol dependence and abuse (in the present study only personal history was considered), since both personal and family histories of alcohol dependence have been shown to affect the antidepressant response to NMDA receptor antagonism (Petrakis et al., 2004; Phelps et al., 2009).

Overall, the data reported here suggest that memantine produces an early anxiogenic response in the emotion-potentiated startle similar to that seen in studies considering a single dose of the SSRI citalopram. However, this is in the absence of any other significant differences in performance on emotional and non-emotional information processing. The profile of effects reported here is very much more limited than that which might be expected in response to a conventional antidepressant agent. There were no effects on the facial expression recognition task or the dot probe task, and whilst there was a marginal effect on emotional recognition memory there were no effects on recall memory. The lack of significant effects on these tasks cannot be explained simply by the fact that this type of task battery is sensitive only to drugs which potentiate serotonin or noradrenaline, as similar tasks have been shown to be affected by treatments with diverse mechanisms of action such as agomelatine (Harmer et al., 2011), high density ion treatment (Malcolm et al., 2009) and vagus nerve stimulation (Critchley et al., 2007).

This more limited profile of effects of memantine is in line with the clinical trial data where memantine was not significantly more effective than placebo in treating depression (Zarate et al., 2006a). As such, the data reported here may reconcile the divergent preclinical and clinical findings, but a subchronic (seven day) study would be useful to confirm this profile of effects. Some animal studies have suggested that memantine may potentiate the effects of conventional antidepressants (Rogoz et al., 2002), and the effect of combination treatment would be worth exploring in emotional processing studies and clinical trials.
